# Fast screening of covariates in population models empowered by machine learning

**DOI:** 10.1007/s10928-021-09757-w

**Published:** 2021-05-21

**Authors:** Emeric Sibieude, Akash Khandelwal, Jan S. Hesthaven, Pascal Girard, Nadia Terranova

**Affiliations:** 1 School of Basic Sciences, Ecole Polytechnique Fédérale de Lausanne (EPFL), Lausanne, Switzerland; 2 Merck Institute for Pharmacometrics (an affiliate of Merck KGaA, Darmstadt, Germany), Merck Serono S.A, Lausanne, Switzerland; 3 Merck Healthcare KGaA, Darmstadt, Germany; 4 Chair of Computational Mathematics and Simulation Science (MCSS), Ecole Polytechnique Fédérale de Lausanne (EPFL), Lausanne, Switzerland

## Abstract

One of the objectives of Pharmacometry (PMX) population modeling is the identification of significant and clinically relevant relationships between parameters and covariates. Here, we demonstrate how this complex selection task could benefit from supervised learning algorithms using importance scores. We compare various classical methods with three machine learning (ML) methods applied to NONMEM empirical Bayes estimates: random forest, neural networks (NNs), and support vector regression (SVR). The performance of the ML models is assessed using receiver operating characteristic (ROC) curves. The F1 score, which measures test accuracy, is used to compare ML and PMX approaches. Methods are applied to different scenarios of covariate influence based on simulated pharmacokinetics data. ML achieved similar or better F1 scores than stepwise covariate modeling (SCM) and conditional sampling for stepwise approach based on correlation tests (COSSAC). Correlations between covariates and the number of false covariates does not affect the performance of any method, but effect size has an impact. Methods are not equivalent with respect to computational speed; SCM is 30 and 100-times slower than NN and SVR, respectively. The results are validated in an additional scenario involving 100 covariates. Taken together, the results indicate that ML methods can greatly increase the efficiency of population covariate model building in the case of large datasets or complex models that require long run-times. This can provide fast initial covariate screening, which can be followed by more conventional PMX approaches to assess the clinical relevance of selected covariates and build the final model.

**Supplementary Information:**

The online version of this article (10.1007/s10928-021-09757-w) contains supplementary material, which is available to authorized users.

## Introduction

Model Informed Drug Discovery and Development (MID3) which often includes Pharmacometrics (PMX) components/elements/aspects, aims to apply mechanistic mathematical and statistical models to pre-clinical and clinical data to efficiently make data-driven decisions during discovery and development phases of new therapies [1]. MID3 plays a key role in each step of drug development [2] by, for example, providing a better understanding of the individual patients’ concentration and response profiles [3], characterizing the drug dose-exposure-pharmacodynamic-response relationships [4], assessing the influence of intrinsic and extrinsic factors on observed intra-individual and inter-individual variability on the considered outcomes [5], and providing predictions of scenarios using different dosing regimens or drug formulations [6]. Among the techniques used in MID3, non-linear mixed effects (NLME) population modeling is an established framework frequently used in pharmacokinetics (PK) and pharmacodynamics for studying variability between individuals in a population by accounting for fixed and random effects [7, 8]. Two important sources of variability considered in the statistical model are the inter-individual variability, which is represented by the variability of a parameter across individuals, and the intra-individual variability considering, for example, the inter-occasion variability of a parameter for a given individual. As part of population model building, relationships between parameters and covariates (each patient’s intrinsic and extrinsic factors) are assessed to explain the inter-individual variability and inform potential dosing recommendations. Assessing the clinical relevance of only the statistically significant covariates is the classical approach as opposed to recent and more computer-intensive full fixed effect model (FFEM) [9, 10] or full random effect model (FREM) [11] approaches, which propose keeping and assessing all covariates in the model.

PMX tools such as non-linear mixed effects modeling (NONMEM) [12], through Pearl-Speaks-NONMEM (PsN) [13, 14] modules, and Monolix [15] provide a wide range of selection-based methods for covariate assessment, including stepwise covariate modeling (SCM) [16], linearization-based methods [17], least absolute shrinkage and selection operator (LASSO) [18], and conditional sampling for stepwise approach based on correlation tests (COSSAC) [19]. However, these methods are not designed to handle multi-dimensional problems involving a variety and a large number of covariates, which is becoming a common situation in the new digital age of medicine in which a vast amount of various types of data are collected. This raises the challenge of finding appropriate and efficient approaches to allow fast screening of large sets of covariates for next-generation PMX and MID3. When supported by a question-driven rationale, the integration of existing methods from other fields can offer unprecedented opportunities to advance methodological frameworks and enhance scientific understanding [20, 21].

In this context, supervised machine learning (ML) provides a large class of algorithms that could be employed for efficient high-dimensional screening of covariates. Among well-known methods, random forest (RF) for classification or regression (2001) [22] and support vector machine (SVM, 1992) [23] have been developed and further improved. More recently, the progress in performance of central processing units (CPUs) and graphical processing units (GPUs) has enabled the development of methods requiring millions of operations, such as deep neural networks (NNs) with complex architectures. This class of new methods is known as deep learning (DL). ML methods are currently employed in various fields and for different purposes. Among applications in medicine, multi-class classification has been raised as a useful tool for disease diagnosis [24], and an increasing number of medical devices using artificial intelligence to diagnose patients more precisely and to treat them more effectively have been qualified by health authorities [25]. ML applications can also be found in all stages of drug development and discovery, including high throughput virtual screening [26], identification of the targeted biomarkers for a given disease [27], *de novo* design of new molecules [28], and assignment in the Biopharmaceutics Drug Disposition Classification System [29]. The adoption of ML into MID3 applications is still in the early stages and a subject of debate within the PMX community [30]. Published examples of ML applications in MID3 are supportive of successful improvements of PK and drug effect predictions [31], longitudinal analyses of tumor size [20, 21], and time to event and survival analysis [32]. Furthermore, population model building could greatly benefit from the integration of methods that could be used to efficiently rank and select relevant covariates based on their importance [33, 34]. In this study, we use and evaluate a few ML approaches for covariate screening and compare the results to traditional PMX selection-based methods in terms of accuracy and computational costs.

## Methods

The performance of ML and PMX methods under various conditions is assessed across scenarios differing in the complexity level of the definition of the true model. Each true model has a predefined number of covariates with a certain correlation level among them and effect size to generate simulated data.

**Fig. 1:**
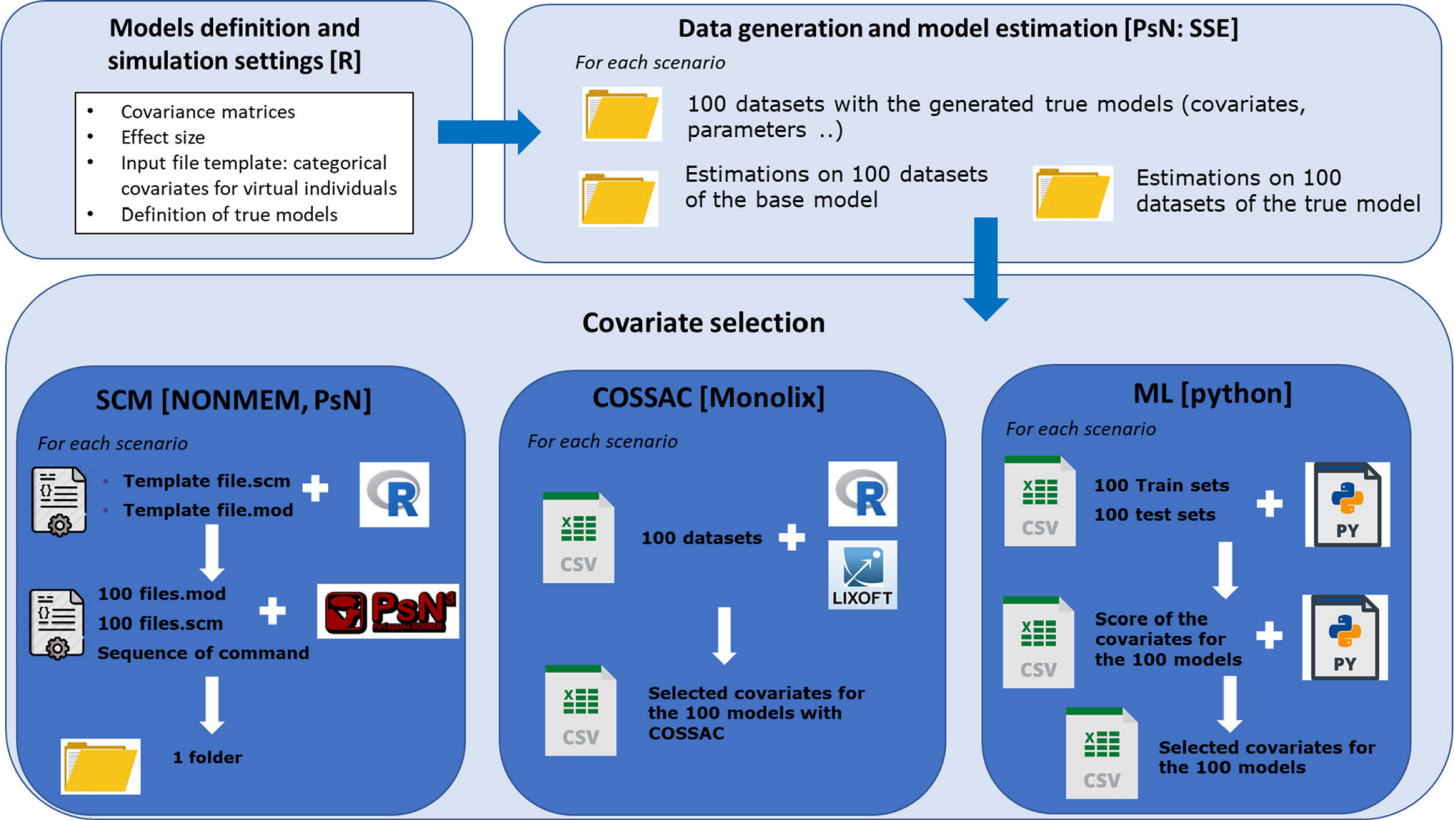
Global project framework. Three main blocks: (i) model definition and simulation settings [R] based on NONMEM templates, covariance matrices, and effect size generation; (ii) data generation and model estimation [PsN: SSE] to simulate data and estimate the base and true models for each scenario (and each dataset); (ii) covariate selection using various methods

The analysis workflow from data simulation to covariate selection is shown in Fig. 1. Model definition and simulation settings are defined in a first step to generate a global framework, allowing the automatic execution of subsequent steps. For each scenario, PK profiles are then generated for a virtual population based on a true model, which is also estimated along with the corresponding base model (i.e., the same model without covariates included). Covariate selection methods available in NONMEM and Monolix are assessed as classical PMX approaches. ML methods include RF, SVR, and NN.

### Use cases

The base model is a one-compartment PK model for single oral administration with first order absorption and linear elimination. A bioavailability of 1 is assumed. The PK parameters are assumed to be log-normally distributed and the exponential error is used for the observed concentration. The PK profile for an individual *i* can be described as follows:

1 $${C}_{i}(t,{V}_{i},{Cl}_{i},{ka}_{i}) = \frac{D\cdot{{ka}}_{i}}{{V}_{i}\cdot {{ka}}_{i} - {{Cl}}_{i}}\cdot({e}^{{t \cdot Cl}_{i}/{V}_{i}}-{e}^{{ka}_{i} \cdot t})$$

where

$${V}_{i} = {V}_{pop}\cdot {e}^{{\eta }_{1,i}}$$

$${CL}_{i} = {CL}_{pop}\cdot {e}^{{\eta }_{2,i}}$$

$${ka}_{i} = {ka}_{pop}\cdot {e}^{{\eta }_{3,i}}$$

The true model was then derived for each scenario by including scenario-specific effects of covariates on clearance (CL). Power and linear relationships are assumed for continuous and categorical covariates, respectively. For one categorical (CAT) and one continuous (COT) covariate, the CL of an individual *i* can be expressed as:

2 $${CL}_{i} = {CL}_{pop}\cdot {e}^{{\eta }_{2,i}}\cdot (1 + CA{T}_{i}\cdot {\beta }_{1})\cdot {\left(\frac{CO{T}_{i}}{med\left(CO{T}_{i}\right)}\right)}^{{\beta }_{2}}$$

The parameters of the structural model are published in a previous simulation study [35]: *V = 10, CL = 1, ka = 1*, ${\omega }_{ka}^{2}=0.3, {\omega }_{V}^{2}=0.2,$ and ${\omega }_{CL}^{2}=0.2$. The variance of the exponential error is set to 0.15.

To better assess a method’s ability to identify the actual covariate part of the true model, a certain number of “false” covariates that do not influence the drug clearance are generated in each scenario. Covariates with and without an effect are referred to as “true” and “false”, respectively. As shown in Table 1a, six combinations are tested. For each of these scenarios, then various levels of effect size (< 20 % or > 40 %) and correlation (small, medium and high) between true-true and true-false covariates, as shown in Table 1b, are assessed. A total of 36 scenarios are studied with covariance matrices and effect sizes randomly generated according to scenario requirements.

**Table 1:** A total of 36 scenarios differing in number of covariates, levels of correlation, and effect size were investigated. For example, the true model of scenario 2e included two categorical and two highly correlated continuous covariates (correlations greater than 0.6), with small effect sizes (< 20 %) of the true continuous (COT) and true categorical (CAT) covariates

(a) Six scenarios with different numbers of true categorical (CAT), true continuous (COT), false categorical (CAF), and false continuous (COF) covariates were tested				
Scenario	#CAT	#COT	#CAF	#COF
Scenario 1	0	1	1	1
Scenario 2	1	1	1	1
Scenario 3	1	1	3	5
Scenario 4	1	2	3	5
Scenario 5	1	3	5	10
Scenario 6	2	2	5	10

(b) Six combinations (a to f) of effect size and correlation for the continuous covariates were studied for all scenarios		
Correlation/Effect	Small (< 20 %)	High (> 40 %)
Small (< 0.3) Medium (> 0.3, < 0.6) High (> 0.6)	a c e	b d f

Such scenarios have an average shrinkage of 5 % (1–30 %), but later two additional scenarios with higher shrinkage are explored. Scenario 7a and 7b are same as scenario 6e except that shrinkage values are 36 % (32–39 %) and 51 % (39–65 %) respectively. The higher shrinkage is induced by reducing the number of observations per subject, as described hereafter.

### Data generation for tested scenarios

Categorical covariates, covariance matrices, and effect sizes for the true covariates (COT and CAT) are generated in the first step (Fig. 1) using R version 3.5.1. [36]. One hundred virtual populations are generated from the true model in each scenario. A design with 5 sampling times per patient is considered: (0 h, 1 h, 8 h, 12 h, 24 h) for half of the patients and (0 h ,2 h, 4 h, 20 h, 48 h) for the other half of the patients. For scenarios with higher shrinkage, the following time grids are used: [0 h, 1 h, 8 h] and [0 h, 2 h, 4 h, 20 h] for scenario 7a; [0 h, 1 h, 8 h] and [0 h, 2 h, 4 h] for scenario 7b. The base model and true model are also estimated in this step using the PsN stochastic simulation and estimation (SSE) module [37].

### Covariate selection in PMX

PsN provides different approaches to perform covariate selection for mixed-effect models, such as SCM and LASSO. SCM is a two-step process in which covariate relationships are tested for forward inclusion and backward elimination based on predefined p-values. PsN allows testing of the linear relationship of categorical covariates, and linear, piecewise, exponential, and power relationships for continuous covariates. In this work, p-values of 0.05 and 0.01 are used for the forward and backward paths, respectively. LASSO allows covariate selection by adding an additional constraint to the parameter space, and then a penalty term to the objective function. The optimization leads to the removal of covariates with a smaller effect in the considered parameter space from the model.

In addition, Monolix supplies the COSSAC algorithm, a variant of SCM that is computationally more efficient. Indeed, only covariates with significant parameter-covariate Pearson correlations are tested in COSSAC. Covariates are then added to the model based on the likelihood ratio test. The algorithm iterates until no forward addition or backward deletion is retained, or after testing 10 new relationships that end without acceptance on the same model. To make the selection faster, Lixoft Monolix developers advise running a first iteration of the stochastic approximation for model building algorithm (SAMBA), as this can provide a starting model much closer to the best model than a base model. This preliminary SAMBA step was always performed in this analysis. To allow method comparisons, the same p-values as in SCM were used for COSSAC. Furthermore, power and exponential relationships for continuous covariates, and linear relationships for categorical covariates can be tested in Monolix.

### Machine learning algorithms

For the covariate selection problem, ML methods: RF, SVR, and NNs, are employed to predict clearance estimates from the base model by starting from all covariates; these are then ranked based on their predictive ability according to the estimated feature importance. Empirical Bayes estimates (EBEs) of clearance obtained in NONMEM were used for this purpose.

**Fig. 2:**
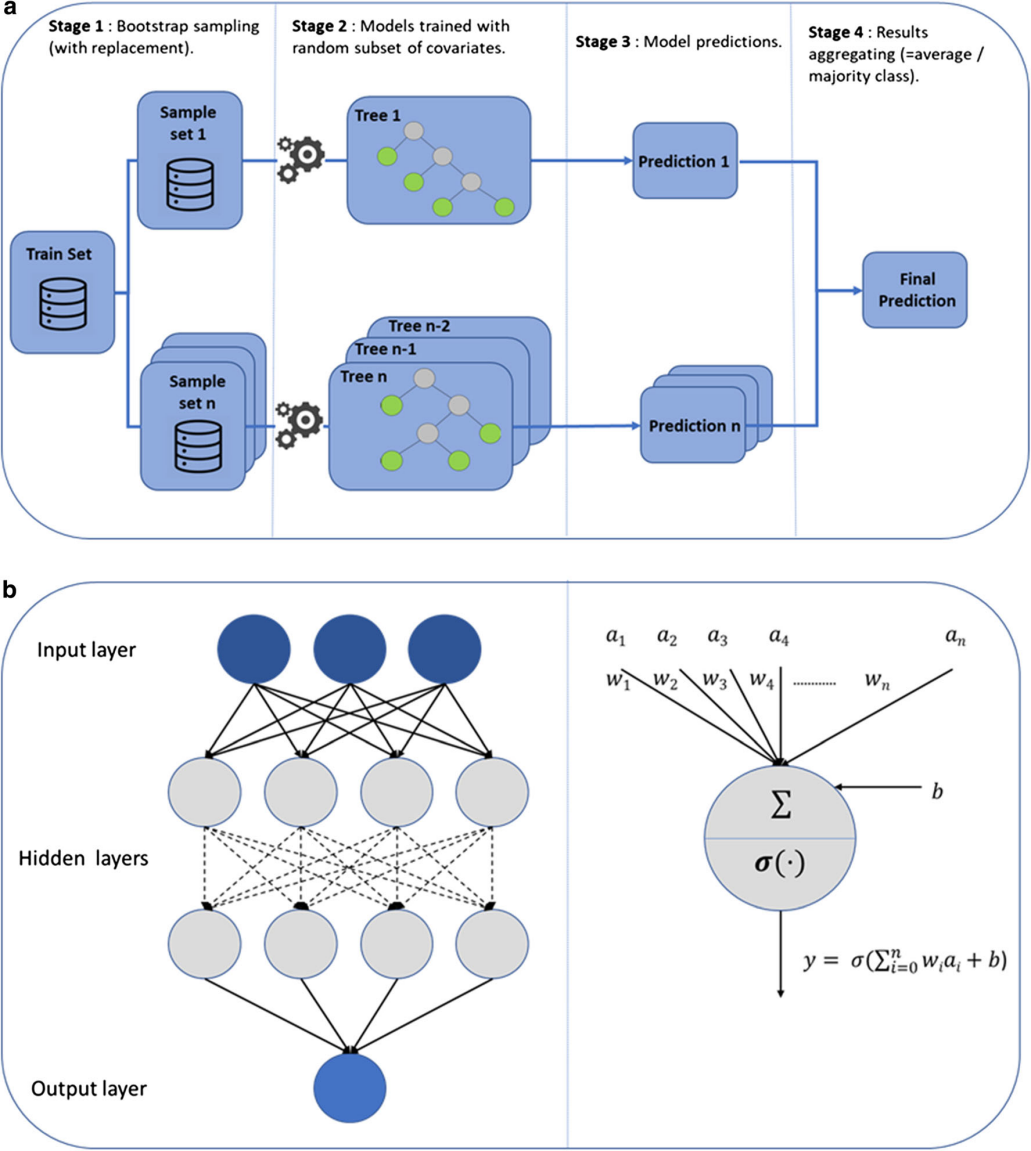
Illustrative examples of two well-known ML methods, random forest (RF) and neural networks (NNs). (a) RF. Stage 1: *n* bootstrap samples with replacement are created. Stage: *n* decision trees are trained. Stage 3: predictions are made from all trees in Stage 2. Stage 4: the final prediction is obtained by averaging all *n* predictions. (b) Simple NN architecture with two hidden layers (left). At each node (right) the information is processed using weights (w) on the inputs and the applying a nonlinear transformation (activation function σ)

RF is an ensemble learning method for classification, regression, and other tasks that combines many decision trees in the training phases and provides the class that is the mode of the classes (classification) or the mean prediction (regression) of the individual trees as output. RF overcomes the limitations (e.g., overfitting) of decision trees by using bagging and feature randomness when building each individual tree. The accuracy of the tree is estimated based on the subset of training data not used in the learning phase, also known as the out-of-bag set. Different hyper parameters have to be set before the training: the number of trees, which is a cost-accuracy trade off, the number of observations in a leaf, and the size of the randomly selected sampling of covariates at each split of a tree, which have to be validated (Fig. 2).

SVM and SVR are supervised learning models used for classification and regression analysis, respectively. Such tasks are similarly handled by implicitly mapping inputs into augmented feature spaces induced through a kernel function. Commonly used kernels are linear, radial basis functions (RBF), or polynomials. The kernel and ε-intensive loss function used to optimize the generalization bounds given for regression are the hyper parameters to be validated before the training phase.

NNs are systems composed of connected units or nodes that mimic neurons in the human brain. Each connection can transmit a signal to other nodes, which process and combine it with their internal state (activation) and an optional threshold through an activation function, and produce output using an output function. Nodes can be grouped into different blocks, the so-called layers, which are sequentially linked in the simplest NN structures to have input, hidden, and output layers. Inputs in the input layers are external data, whereas output layers give the estimated outputs. The optimization process involves a forward path, a backward path, and a gradient descent-based optimizer (e.g., ADAM [41], AdaGrad, stochastic gradient descent [42]) to update network parameters. For the prediction part, only the forward path is used with the weights optimized during the training phase. Potential NN overfitting is overcome by using regularization techniques, such as dropout [43], which is a model-averaging method randomly removing some weights based on a prior probability. NN hyper parameters to be set (and ideally validated) before the training include the number and size of hidden layers, type of activation function for each node, and the number of iterations for the gradient descent algorithm. Flexibility is the NNs’ main strength, but it could also be a substantial drawback. It allows broad application by predicting any kind of outcome and by accepting various input data, such as images or text-based features, without any pre-processing. However, NNs can lead to a very large number of different architectures and uninterpretable models when, in many cases, the interpretation of the results can be as important as the accuracy of the predictions (Fig. 2).

### Hyper-parameters in ML

Many ML methods require setting a few hyper-parameters before the training phase, as these cannot be learnt from the data and remain the same throughout the analysis. Some have been mentioned in the previous section for each considered method. In this study, the same hyper parameters were used in all 100 models trained in each scenario. This assumption, which allows real computational gain, is supported by the 100 training sets being independently and identically distributed. The learning phase is achieved using the mean square error (MSE) as the cost function.

For RF, the number of trees was set to 500 for all scenarios and datasets. For each scenario, a grid search is performed to select the number of observations in a leaf (from 1 to 50) and the ratio of covariates randomly selected at each split of a tree (from 0.3 to 0.7). For SVR, two different kernels are tested: linear and RBF. The regularization parameter and ε are validated using a grid search ranging from 10^− 5^ to 1 and from 10^− 4^ to 1, respectively.

For NNs, no grid search is performed and only architectures with two hidden layers are tested. The best architecture includes 500 neurons, ADAM optimizer, and dropout probability of 0.3 to avoid overfitting. The chosen activation function is ReLU (truncated identity). After selecting the hyper parameters for each scenario, models are independently trained on all datasets for all methods.

### Importance score and covariate selection

The method for calculating the importance score, available by default in RF, is also used for SVR and NN. Specifically, for each covariate, the “resampling error” after covariate shuffling is calculated and compared to the validation error (or the out-of-bag error for RF). The more important the covariate, the greater is their difference. Error differences are rescaled to have the sum over the covariates equal to 1. This rescaling leads to the importance score. Covariate shuffling is performed 100 times to decrease the variance of the results. The resampling is done using a different validation set than the one used for training.

The literature advises human intervention to perform covariate selection from the importance score. Ideally, a large difference between the importance scores of two successive covariates allows clear separation of covariates by their importance. However, given the number of datasets in our study (n = 3600), automatic selection is implemented as being more efficient. Specifically, three selection approaches using the importance score are investigated: top-M selection, order of importance, and minimum degree of importance. Top-M selection consists of ranking the covariates by importance (according to their score) and then keeping only a predefined number (*M*) of the most important covariates. In the “order of importance” approach, covariates are ranked by order of importance and then selected until the sum of scores of selected covariates reaches a predefined threshold. Finally, in the “minimum of importance” approach, only covariates with an importance score greater than a predefined threshold are selected. All three methods require setting of a predefined parameter (*M* or a threshold). For each scenario and each method, a wide range of thresholds were tested and the threshold maximizing the accuracy of selection kept. In the case of top selection, *M* is set to the number of true covariates with the assumption that an accurate method ranks them on top.

### Metrics for evaluation

The discriminatory performance of ML approaches/models is assessed by using the receiver operating characteristic curve (ROC), a probability curve representing the true positive rate (rate of true outcomes correctly predicted as true) against the false positive rate (rate of false outcomes wrongly predicted as true). After computing the ROC, the area under the ROC (AUROC) can be calculated. The AUROC provides a measure of the degree of separability of the classes predicted from the ML approaches. Its values can be categorized into “excellent” (0.9–1), “good” (0.8–0.9), “fair” (0.7–0.8), “poor” (0.6–0.7), or “failed” (0.5–0.6) outcomes [44]. An AUROC of 0.5 corresponds to a random guess; therefore, AUROC < 0.5 indicates that a method performs worse than a random guess. In order to compute the AUROC, the ROC must be drawn. This is achieved by performing several selections and deriving the respective true positive and false positive rates. In this work, ROCs are derived for all ML methods by increasing the threshold from 0 to 1 for the order of the importance selection approach described previously. The AUROC can be used only for comparing the results obtained from ML methods. Indeed, as SCM, COSSAC, and LASSO provide only one covariate selection for parameter settings, ROCs generation would not be very practical for these PMX methods. For this reason, the F1 score (the harmonic mean of recall and precision) was computed (3) and used to compare selection results between ML and PMX methods.

3 $$F1 = 2 \cdot\frac{Recall\cdot Precision}{Recall +Precision}$$

where$$Recall = \frac{TruePositive}{TruePositive +FalseNegative}$$ and $$Precision =\frac{TruePositive}{TruePositive + FalsePositive}$$

The highest possible value of the F-score is 1, indicating perfect precision and recall, and the lowest possible value is 0, if either the precision or the recall is zero [45]. The F1 score is more robust with imbalanced classes than the accuracy metric, defined as ($$TruePositive +TrueNegative)/Number of Predictions$$. In the case of SCM and COSSAC, two definitions of true positive are applied and used to compute the F1 score: true covariate selected, and true covariate selected with the true relationship. As only linear relationships can be tested for categorical covariates, no other definitions are applied. Results obtained with the two definitions for the two approaches are hereafter referred to as SCM and SCM_TR and as COSSAC and COSSAC_TR, respectively.

### Real case

The developed framework is also tested on a clinical dataset to illustrate its use in a real case study. First, the base model is estimated using a PMX software (NONMEM in this case), and then a fast screening of covariates is performed using the considered methods. The use case includes data from a recently published study on cetuximab, and described by a two-compartment model with Michaelis-Menten and linear elimination [46] as the base model. Thirty covariates are available in the dataset, including patient-related factors (e.g., age, sex, creatinine clearance), therapy related-factors (e.g., co-medication), and other disease-related measurements (e.g., amphiregulin, interleukin-8). All covariates are included in the selection step; no clinical or statistical assessments are available from previous analysis.

### Software

NONMEM (7.3.0) installed on LINUX (Novell SLES11 (64-bit) SP3) operating system, with CPU allocation controlled by a Univa Grid Engine (8.2). The NONMEM runs along with SCM evaluations are executed by PsN (4.4.8). COSSAC selection requires Monolix 2019R2 which is installed on a different server with the same LINUX operating system. The ML part is implemented using Python (3.7, [38]) along with scikit-learn (0.22.1, [39]) and Pytorch (1.3.1, [40]) installed on Anaconda 1.9.12.

## Results

### Covariate selection

#### Assessment of AUROC across ML methods

**Fig. 3:**
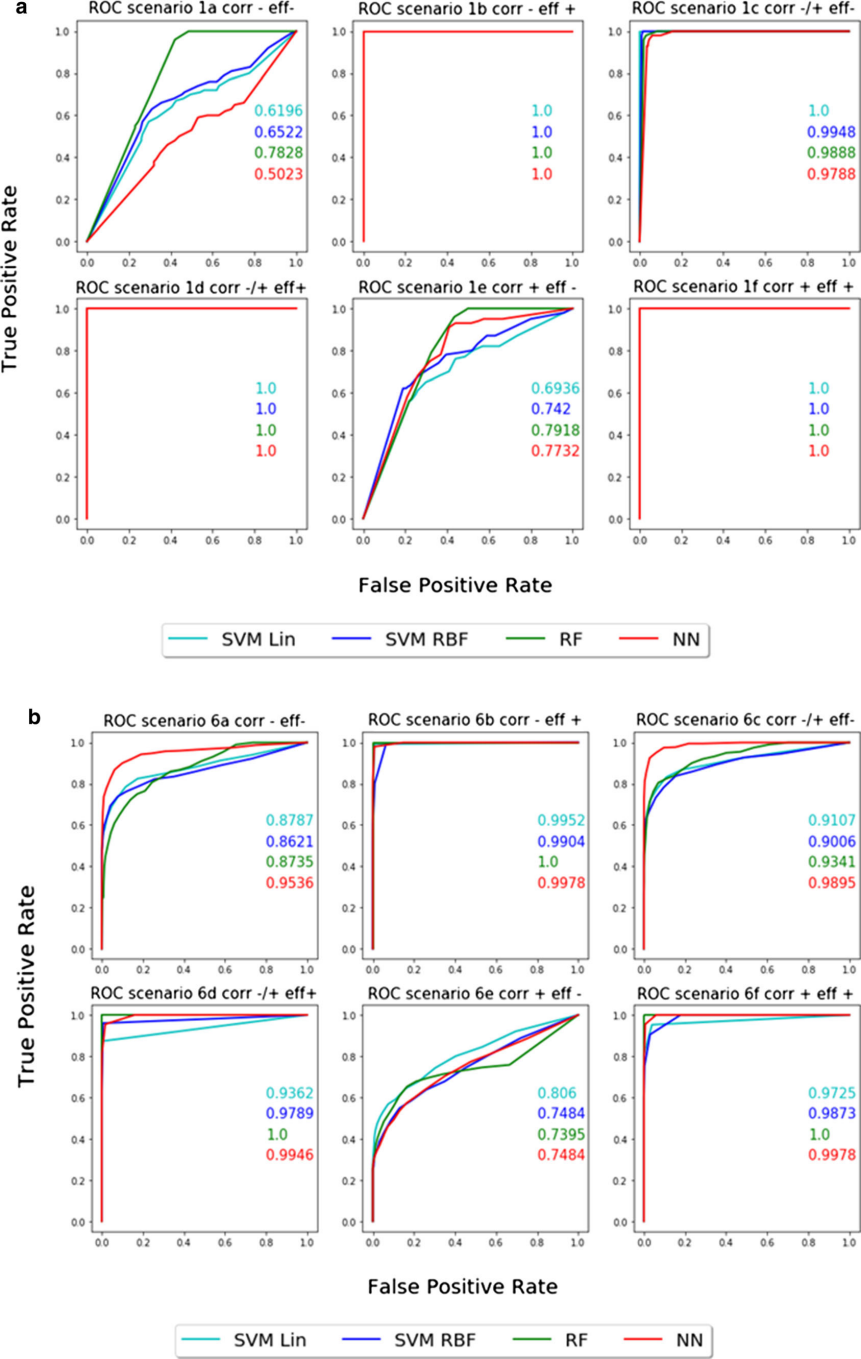
ROC and AUROC of the four ML methods: RF (green), NN (red), SVR with linear kernel (cyan), and SVR with RBF kernel (blue) for two scenarios. (a) Scenario 1:1 true continuous covariate and two false covariates (1 continuous and 1 categorical). (b) Scenario 6: 2 continuous true, 2 categorical true, 10 continuous false and 5 categorical false covariates. The different levels of effect size (eff) and correlation (corr) are given as small (-), medium (-/+), and high (+). Similar performances were observed across methods

Similar results in terms of AUROC were obtained across all ML methods, with NNs showing slightly better performance. Examples are shown in Fig. 3 of the ROCs and AUROCs obtained in scenarios 1 and 6 across models. The effect size of covariates is the most important factor influencing results. In the case of high effect size (scenarios *b*, *d*, *f*), the importance scores for any true covariate are always greater than the importance score for any false covariate for all 100 datasets, leading to perfectly separable classes (AUROC = 1). Significantly less precise selections are obtained for some scenarios with low effect size (scenarios *a*, *c*, *e*). For example, in scenario 1*a*, the AUROCs for NN and SVR are very close to a random guess. There are no indication of an impact of correlation between the covariates. Observed fluctuations in AUROCs between scenario 1*a*, 1*c*, and 1*e* are due to changes in effect size during their random generation.

The number of overall false covariates, which can be seen as noise, does not impact the ability of ML to correctly identify true and false covariates in considered scenarios. Overall, AUROCs obtained by the ML methods are very good and > 0.90 on average (Online Resources Fig. S1) with perfect separation (AUROC = 1) in several cases (18 scenarios by RF, 11 scenarios by NN, 12 and 11 scenarios by SVR linear and SVR RBF, respectively).

#### Assessment of F1 score across ML and PMX methods

The goodness of covariate selection results can be evaluated for both ML and PMX by computing the F1 score. In Table 2, average F1 scores with their standard deviation are reported for all methods, along with the number of scenarios achieving best results (F1 score = 1). Detailed results are also reported in Online Resources Table S1 and S2. Overall, similar performance is achieved across ML methods, which slightly outperforms SCM or has similar accuracy. SCM and COSSAC provided similar results. However, no PMX approach provides perfect selection for all 100 datasets in any scenario. As expected, because of their stricter definition of true positive, SCM_TR and COSSAC_TR are less accurate than SCM and COSSAC and are often outperformed by all other methods. For some scenarios (e.g., scenario 1*a* and 1*e*), the F1 scores were very small (~ 0.2).

As covariate selection in SCM is based on p-values chosen in the forward and backward path, scenarios with worse selection (1*a*, 1*e*, 5*a*) are reassessed with different p-value combinations (see Online Resources Table S***3***). Changing the p-value in the backward path has no effect on the selection, whereas increasing the p-value in the forward path increases the accuracy of the selection in scenarios 1*a* and 1*e*. F1 scores do not improve for scenario 5a.

Overall, F1 scores obtained from SCM remain lower than those obtained with the ML methods.

The best method is NN on average, but RF is the method with the greatest number of perfect selections regardless of the selection approach. As shown in Table 2, LASSO leads to the least accurate results, likely due to the LASSO implementation in PsN allowing only linear relationships to be tested, hence limiting identification of the true relationship for continuous covariates.

**Table 2:** Summary of the main results for the ML methods and PMX methods. For each method, the number of scenarios in which the method achieved perfect selection of the covariate (first row), and the average F1 score with standard deviation in brackets (second row) are reported. For ML methods, results with the three considered approaches (top-M, order of importance, minimum of importance) are shown. TR: True

ML approaches			
Method\Selection	Top-M	Order of Importance	Minimum of Importance
RF	♣ 18 scenarios ♣ 0.87 (0.16)	♣ 18 scenarios ♣ 0.88 (0.15)	♣ 16 scenarios ♣ 0.88 (0.15)
NN	♣ 16 scenarios ♣ 0.89 (0.16)	♣ 8 scenarios ♣ 0.88 (0.15)	♣ 14 scenarios ♣ 0.89 (0.15)
SVR Linear	♣ 15 scenarios ♣ 0.86 (0.16)	♣ 11 scenarios ♣ 0.85 (0.15)	♣ 13 scenarios ♣ 0.87 (0.15)
SVR RBF	♣ 16 scenarios ♣ 0.86 (0.17)	♣ 9 scenarios ♣ 0.85 (0.16)	♣ 13 scenarios ♣ 0.87 (0.16)
PMX approaches			
SCM	♣ 0 scenario ♣ 0.84 (018)		
SCM_TR	♣ 0 scenario ♣ 0.75 (0.19)		
COSSAC	♣ 0 scenario ♣ 0.79 (0.016)		
COSSAC_TR	♣ 0 scenario ♣ 0.72 (0.18)		
LASSO	♣ 0 scenario ♣ 0.65 (0.18)		

### Computational costs

The runtimes of the methods are reported for scenarios with high (including more than 15 covariates) and low (including less than 5 covariates) complexity and for all scenarios in Table 3. NN, SCM and COSSAC methods allow parallelization of runs with multiple CPUs (12 for NN and SCM, 8 for COSSAC), leading to reduced runtimes, whereas only a single CPU can be used with RF and SVR. This difference is not inherent to the methods, but to the way they are implemented in the tools and packages that were used.

**Table 3:** Computational cost (in hours) of considered methods. Costs are reported as average values with standard deviation in brackets across all scenarios, for complex scenarios (including more than 15 covariates: 5a-f, 6a-f), and for simple scenarios (including less than 5 covariates: 1a-f, 2a-f)

	SCM	COSSAC	SVR Lin	SVR RBF	RF	NN
Simple Scenario	9.5 (4.08)	3.6 (1.7)	0.08 (0.06)	0.13 (0.09)	0.10 (0.02)	2.3 (0.29)
Complex Scenario	87.6 (33.0)	13.0 (3.3)	0.55 (0.16)	0.68 (0.13)	0.16 (0.08)	7.75 (026)
Overall	27.1(40.3)	7.4 (5.2)	0.3 (0.2)	0.41 (0.2)	0.12 (0.06)	3.4 (2.4)

Despite the additional computation resources, the SCM runtime exceeds 3 days for some scenarios, whereas ML methods are run in less than 3 h even in the most complex scenarios. On average, ML is at least 8, and up to 225-times faster than SCM. LASSO (not shown) has runtimes comparable to SCM. COSSAC is on average 4-times faster than SCM, but still slower than ML. Moreover, the computational cost ratio between SCM and COSSAC increases with the number of covariates, suggesting that, compared to SMC, only COSSAC is able to handle many covariates. Focusing on ML methods only, the SVR runtime increases linearly with the number of covariates due to the resampling of each covariate for the scoring calculation. The NN runtime is balanced between the tasks related to model training and those related to the scoring calculation. RF has the best runtime across all methods caused partly by the default implementation and the optimization of the scoring calculation.

Overall, the ML and PMX methods have very different computational costs, with a clear benefit provided by the ML approaches. Such differences become even more relevant with an increasing number of covariates. Although ML methods require an additional run on PMX software to build the final model, results will not change, as this last model estimation is much faster than model selection, as only a subset of the covariates would be used. Further, the uncertainty of runtimes differs from one method to another in this study. The computational cost of SCM and LASSO is easily computed by assigning a predefined number of CPUs to the task on an external cluster through PsN. For ML methods, deriving the actual computation cost is not straightforward. as methods are locally trained and then runtimes impacted by other programs or competitive tasks. Moreover, the runtimes given for ML include the time spent on the grid-search, which consist of testing 25 to 125 combinations (models) of hyper parameters depending on the methods. Increasing or decreasing the number of such combinations directly impacts the computational cost of the method.

### Real case

The four ML methods are used to estimate the top 10 predictors of cetuximab clearance and the central volume of distribution from the 30 initial covariates. Six of the top 10 covariates for clearance are the same across the methods. Three additional covariates are selected by three methods. For volume, four covariates are selected by all methods and two additional covariates by three methods. Given the parameter estimates of the base model, importance scores for the two parameters are computed in approximately 10 min. No comparison is made with SCM, as runtimes exceed 19 days. This computational difference is greater than the one previously discussed because the base model in this real case is much more complex. While the model complexity greatly impacts SCM, which requires the estimation of many models within each path, this has no effect on ML methods, in which only the base model has to be estimated. Thus, this real use case further highlights the benefit of adopting ML methods for the fast screening of covariates not only when a large set is involved, but also when base models are complex and long estimation runtimes are required.

### Evaluation of EBEs shrinkage

ML methods use EBEs to perform covariate selection, relating the accuracy of the selection to EBEs. Results from the investigation of scenarios 7a and 7b show that the performance of all methods is impacted by the higher shrinkage as indicated by the low F1 scores (Online Resources Table S4). NN performs better than the other methods. Depending on the strategy of selection, RF has similar or better performances than SCM. No differences between SCM and SVM (with both kernels) are suggested. As for other scenarios, F1 scores for COSSAC and SCM are comparable. F1 scores for scenario 7b are slightly higher than those for scenario 7a; this is likely due to number fluctuations. AUROCs for the ML methods are shown in Online Resources Fig. S2.

## Discussion

The integration of ML methods into MID3 problems represents a great opportunity for more efficient and advanced analytical solutions. In this study, the use of different ML algorithms for fast screening of covariates in population models is assessed and compared to state-of-the-art PMX techniques. True models which differ in the number of covariates (1 to 7 categorical, 1 to 12 continuous), correlations between covariates (three levels), and effect size (two levels) were defined and used to assess the methods across a total of 36 scenarios. Two additional scenarios with higher values of EBEs shrinkage are also investigated. The exploration of time-varying covariates was out-of-scope of this analysis.

SCM and LASSO implemented in PsN, and COSSAC in Monolix are compared with four ML methods: RF, SVR with RBF or linear kernels, and NNs. To provide an automatic assessment of results, three selection approaches, based on covariate scores were studied and assessed in terms of AUROC and F1 scores. The AUROCs indicate a perfect covariate ranking in half of the scenarios for RF, and NNs achieve the highest AUROC on average. SVR with both kernels led to similar results. The comparison of ML and PMX methods is based on F1 scores, which indicates slightly better performance for ML methods. In addition, the evaluation of computational costs suggested a significant benefit of ML over standard PMX approaches, with an average runtime of 1 h versus 27 h for SCM and 7.5 h for COSSAC. This benefit remains significant after adding the runtime of a full final model in Monolix or NONMEM. Lower F1 scores are obtained for all methods when assessing their performance in scenarios with the higher shrinkage values.

To further investigate available options to optimize PMX approaches, the GAM method [10] and the linearization-based SCM were tested and compared with the other methods for scenario 6e, representative of most common scenarios. The use of linearization greatly improves the computational cost of SCM (from 73 to 16.3 h), while preserving its accuracy. Such runtime is still larger than those of ML methods (0.25 h for RF, 0.55 h for SVR both kernels, 3.1 h for NN), but comparable to COSSAC (15.7 h). GAM computational cost is of 2.5 h, thus, comparable to NN. However, the F1 score was the lowest across all considered methods (Online Resources Table S5). Of note, the computational gain of SCM and COSSAC could be further increased by reducing the number of tested relationships.

This work highlights the significant contributions that ML methods could offer during an initial fast screening of covariates to inform subsequent population modeling steps. This is especially valid now, as an increasing amount of data is being collected at the population and individual patient levels. These conclusions are validated in an extreme case including 95 false covariates and 5 true covariates. To keep the PsN-NONMEM runtime reasonable, only five datasets were simulated for this scenario. The results are in line with those obtained in the overall analysis. The SCM F1 score is 0.85 and the computation on 12 CPUs takes more than 450 h. ML methods have different F1 scores: 1 for RF, 0.8 for SVR regardless of the kernel used, and 0.68 for NN. Runtime differences were considerable: less than 1.5 h for RF, 3 h for NN on 12 CPUs, 12 h for SVR with a linear kernel, and 10 h for SVR with RBF kernel. With only 11 h of computation and an F1 score of 0.81, COSSAC performs well and is equivalent to ML methods in this extreme scenario.

ML approaches are tested in only one relatively simple structural and statistical model; however, as demonstrated in our real use case, the established framework can be generalized to any model. The computational gain provided by ML methods becomes even larger with complex structural models because ML does not require solving the differential equations, and its performance is not going to be impacted by the model complexity. Using ML on real datasets to increase the efficiency of population PKPD covariate model building in the case of a large data sets or complex models with intensive runtimes could be envisaged by adopting ML for fast initial covariate screening, and then consider the top (e.g., 10 or 20) covariates with higher importance score in a final model-building step, performed according to standard and preferred PMX approaches (e.g., SCM, FFEM, FREM). The PMX step is crucial to end with a satisfying model able to assess the relevance of the covariates and to be usable for prediction in different patient populations and dosing scenarios for an impactful MID3.

The power of ML in obtaining fast and accurate results for large datasets has already been demonstrated over the last decade. The challenge is now to include ML in state-of-the-art processes to improve their performance. Our study highlights the great potential and synergistic effect of integrating these techniques into classical PMX modeling by leveraging their use for the next generation of MID3 in the digital data era.

## Supplementary Information

Below is the link to the Supplementary Information.

Supplementary Information 1 (DOCX 531 kb)

## References

[1] Marshall SF, Burghaus R, Cosson V (2016). Good practices in model-informed drug discovery and development: practice, application, and documentation. CPT Pharmacomet Syst Pharmacol.

[2] Marshall S, Madabushi R, Manolis E (2016). Model-informed drug discovery and development: current industry good practice and regulatory expectations and future perspectives. CPT Pharmacomet Syst Pharmacol.

[3] Derendorf H, Meibohm B (1999). Modeling of pharmacokinetic/pharmacodynamic (PK/PD) relationships: concepts and perspectives. Pharm Res.

[4] Holford N, Sheiner HG, Lewis B (1981). Understanding the dose-effect relationship. Clin Pharmacokinet.

[5] Roden DM, Wilke R, Akroemer H (2011). Pharmacogenomics: the genetics of variable drug responses. Circulation.

[6] Hugh DCH (2003). The influence of formulation variables on the performance of alternative propellant-driven metered dose inhalers. Adv Drug Deliv Rev.

[7] Mould DR, Upton RN (2012). Basic concepts in population modeling, simulation, and model-based drug development. CPT Pharmacomet Syst Pharmacol.

[8] Mould DR, Upton RN (2013). Basic concepts in population modeling, simulation, and model-based drug development—part 2: introduction to pharmacokinetic modeling methods. CPT Pharmacomet Syst Pharmacol.

[9] Gastinguay M (2004). A full model estimation approach for covariate effects: inference based on clinical importance and estimation precision. AAPS J.

[10] Mandema J, Wverotta D, Sheiner LB (1992). Building population pharmacokinetic pharmacodynamic models. I. Models for covariate effects. J Pharmacokinet Biopharm.

[11] Karlsson MO (2012) A full model approach based on the covariance matrix of parameters and covariates. Population Approach Group Europe (PAGE) 21. Abstr 2455

[12] Rjnonmem B (2019). Tutorial part I: description of commands and options, with simple examples of population analysis. CPT Pharmacomet Syst Pharmacol.

[13] Keizer R, Karlsson MO, Hooker A (2013). Modeling and simulation workbench for NONMEM: tutorial on Pirana, PsN, and Xpose. CPT Pharmacomet Syst Pharmacol.

[14] Lindbom L, Ribbing J, Jonsson EN (2004). Perl-speaks-NONMEM (PsN)—a Perl module for NONMEM related programming. Comput Methods Progr Biomed.

[15] Monolix (2018R2) Antony, France: Lixoft SAS, 2018

[16] Jonsson E, Karlsson N, Mats O (1998). Automated covariate model building within NONMEM. Pharm Res.

[17] Khandelwal A, Harling K, Jonsson NE (2011). A fast method for testing covariates in population PK/PD models. AAPS J.

[18] Ribbing J, Nyberg J, Caster O (2007). The lasso—a novel method for predictive covariate model building in nonlinear mixed effects models. J Pharmacokinet Pharmacodyn.

[19] Traynard P, Ayral G, Twarogowska M (2020). Efficient pharmacokinetic modeling workflow with the monolix suite: a case study of remifentanil. CPT Pharmacomet Syst Pharmacol.

[20] Terranova N, Girard P, Ioannou K (2018). Assessing similarity among individual tumor size lesion dynamics: the CICIL methodology. CPT Pharmacomet Syst Pharmacol.

[21] Vera-Yunca D, Girard P, Parra-Guillen Z (2020). Machine learning analysis of individual tumor lesions in four metastatic colorectal cancer clinical studies: linking tumor heterogeneity to overall survival. AAPS J.

[22] Breiman L (2001). Random forests. Mach Learn.

[23] Burges CJC (1998). A tutorial on support vector machines for pattern recognition. Data Mining Knowl Discov.

[24] Liang H, Tsui, Brian Y, Hao NI (2019). Evaluation and accurate diagnoses of pediatric diseases using artificial intelligence. Nat Med.

[25] Liu Q, Zhu H, Liu C (2020). Application of machine learning in drug development and regulation: current status and future potential. Clin Pharmacol Therapeut.

[26] Khandelwal A, Krasowski M, Erica D, Reschly J (2008). Machine learning methods and docking for predicting human pregnane X receptor activation. Chem Res Toxicol.

[27] Jeon J, Nim S, Teyra J (2014). A systematic approach to identify novel cancer drug targets using machine learning, inhibitor design and high-throughput screening. Genome Med.

[28] Haghighatlari M, Hachmnn J (2019). Advances of machine learning in molecular modeling and simulation. Curr Opin Chem Eng.

[29] Khandelwal A, Bahadduri P, Praveen M, Cheng C (2007). Computational models to assign biopharmaceutics drug disposition classification from molecular structure. Pharm Res.

[30] Baker RE, Pena J-M, Jayamohan j (2018). Mechanistic models versus machine learning, a fight worth fighting for the biological community?. Biol Lett.

[31] You W, Widmer N, De Micheli G (2011) Example-based support vector machine for drug concentration analysis. In: 2011 Annual International Conference of the IEEE Engineering in Medicine and Biology Society. IEEE, pp. 153–157 10.1109/IEMBS.2011.6089917 22254273

[32] Gong X, Hu M, Zhao L (2018). Big data toolsets to pharmacometrics: application of machine learning for time-to‐event analysis. Clin Transl Sci.

[33] Talevi A, Morales J, Hather G (2020). Machine learning in drug discovery and development part 1–a primer. CPT Pharmacomet Syst Pharmacol.

[34] Koch G, PfisterM DaunhawerI (2020). Pharmacometrics and machine learning partner to advance clinical data analysis. Clin Pharmacol Therapeut.

[35] Lavielle M, Ribba B (2016). Enhanced method for diagnosing pharmacometric models: random sampling from conditional distributions. Pharm Res.

[36] R Core TEAM (2017) R: a language and environment for statistical computing

[37] Ueckert S, Karlsson Mats O, Hooker AC (2016). Accelerating Monte Carlo power studies through parametric power estimation. J Pharmacokinet Pharmacodyn.

[38] VAN Rossum G, Drake JR, Fred L (1995). Python reference manual.

[39] Pedregosa F, Varoquaux G, Gramfort A (2011). Scikit-learn: machine learning in Python. J Mach Learn Res.

[40] Paszke A, Gross S, Massa F (2019). PyTorch: an imperative style, high-performance deep learning library. Advances in neural information processing systems.

[41] Kingma D, Jimmy A (2014) A method for stochastic optimization. arXiv preHTML arXiv:1412.6980

[42] Ruder S (2016) An overview of gradient descent optimization algorithms. arXiv preHTML arXiv:1609.04747

[43] Frey B, Leung MKK, Delong AT et al (2019) Systems and methods for classifying, prioritizing and interpreting genetic variants and therapies using a deep neural network. U.S. Patent No 10,185,803, 22 Jan 2019

[44] Kleinbaum D, Klein M (2010). Ordinal logistic regression. Logistic regression.

[45] Goutte C, Gaussier E (2005). A probabilistic interpretation of precision, recall and F-score, with implication for evaluation. European conference on information retrieval.

[46] Grisic A-M, Khandelwal AB, Huisinga M, Kloft C (2020). Semimechanistic clearance models of oncology biotherapeutics and impact of study design: cetuximab as a case study. CPT Pharmacomet Syst Pharmacol.

