# Supplementary material for: Fast screening of covariates in population models empowered by machine learning
Source: J Pharmacokinet Pharmacodyn. 2021 May 21;48(4):597–609. doi: 10.1007/s10928-021-09757-w (PMC8225540; doi:10.1007/s10928-021-09757-w)
Supplement: Supplementary file 1 — Supplementary Information 1 (DOCX 531 kb) [file 10928_2021_9757_MOESM1_ESM.docx]

# Online Resources
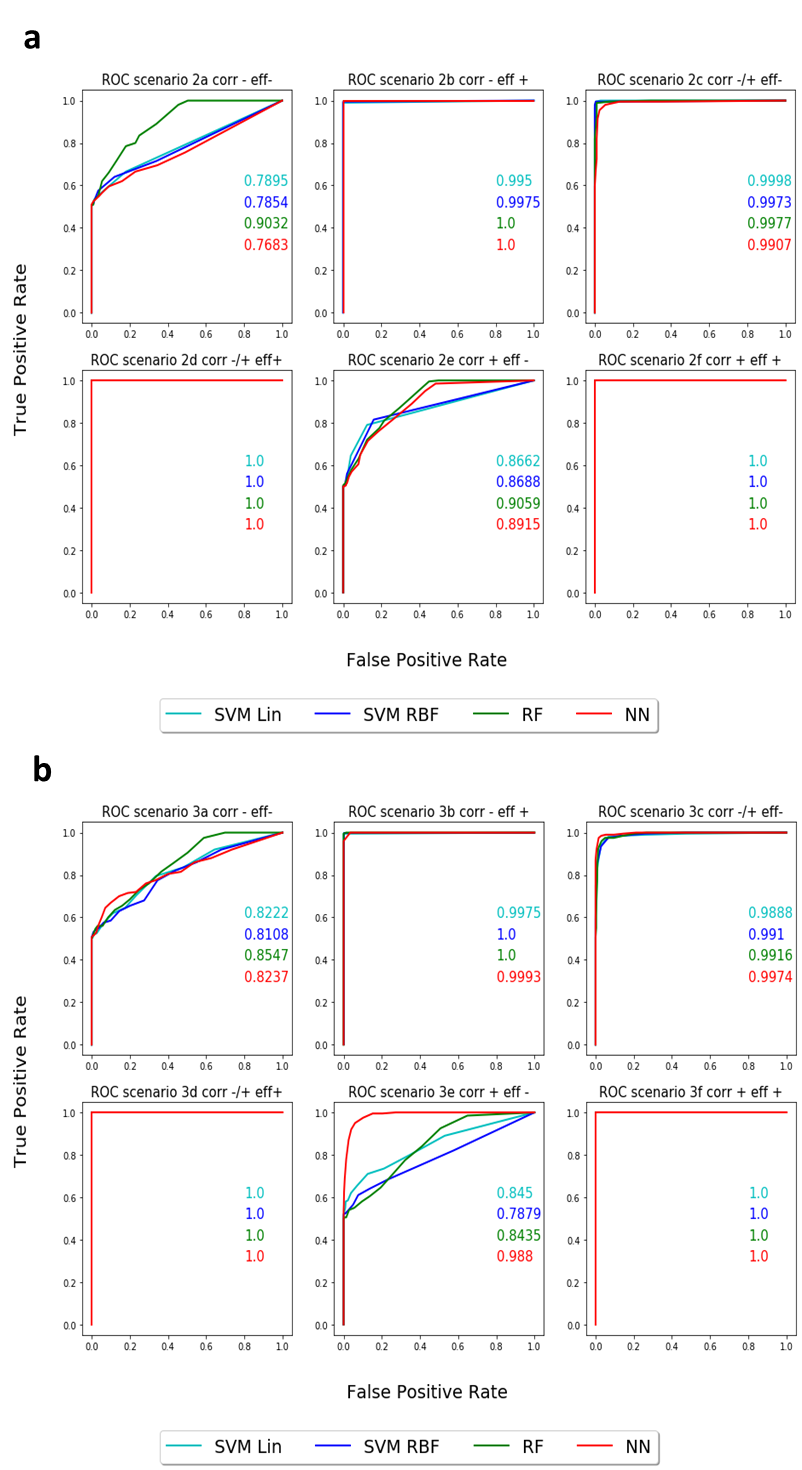

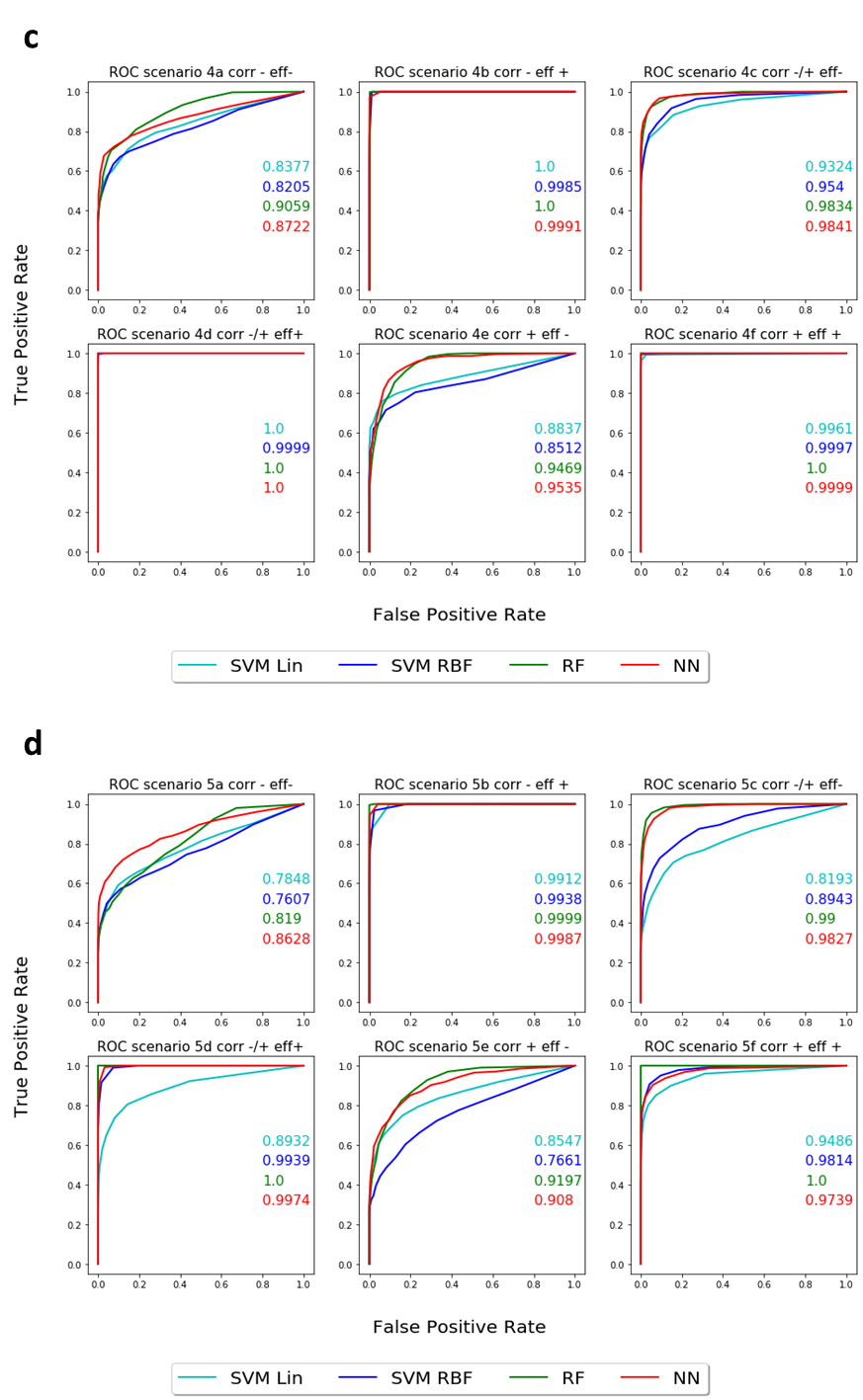


**Fig. S1** ROC and *AUROC* of the four ML methods for scenarios 2 to 5. RF (green), NN (red), SVR with linear kernel (cyan), and SVR with RBF kernel (blue). The different levels of effect size (eff) and correlation (corr) are given as small (-), medium (-/+), and high (+). (a) Scenario 2: 1 continuous true, 1 categorical true, 1 continuous false and 1 categorical false covariate. (b) Scenario 3: 1 continuous true, 1 categorical true, 5 continuous false and 3 categorical false covariates. (c) Scenario 4: 2 continuous true, 1 categorical true, 5 continuous false and 3 categorical false covariates. (d) Scenario 5: 3 continuous true, 1 categorical true, 10 continuous false and 5 categorical false covariates. Similar performances were observed across methods

**
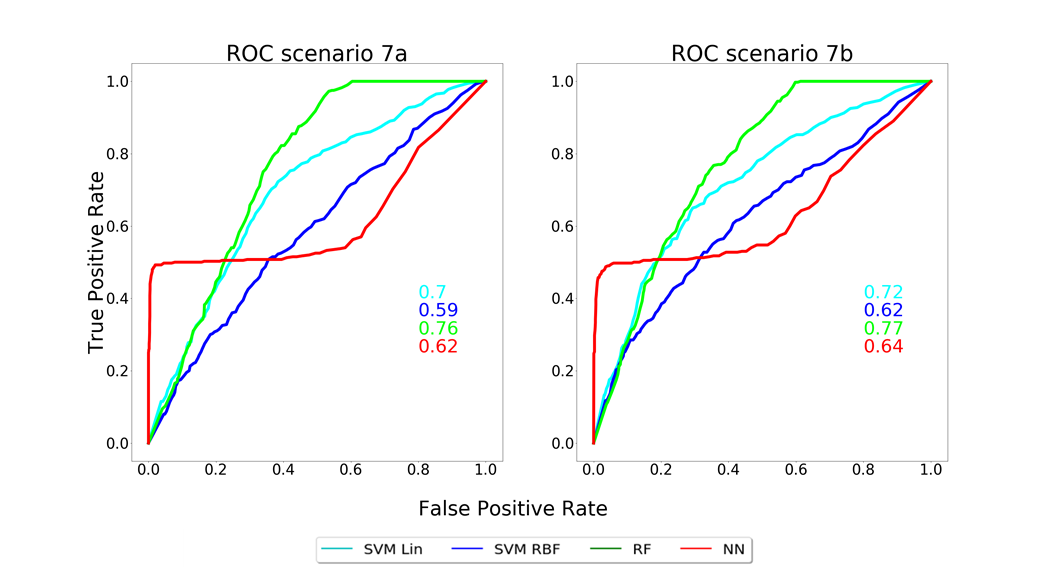
**

**Fig. S2** ROC and *AUROC* of the four ML methods for scenarios 7a and 7b. RF (green), NN (red), SVR with linear kernel (cyan), and SVR with RBF kernel (blue).

| **ML approaches** | | | |
| --- | --- | --- | --- |
| **Method\Selection** | **Top-M** | **Order of Importance** | **Minimum of Importance** |
| **RF** | 1. 18 scenarios 2. 0.87 (0.16) 3. 0.88 (0.17) 4. 0.85 (0.18) 5. 0.74 (0.15) 6. 1.0 (0.0) | 1. 18 scenarios 2. 0.88 (0.14) 3. 0.91 (0.12) 4. 0.85 (0.16) 5. 0.77 (0.12) 6. 1.0 (0.0) | 1. 16 scenarios 2. 0.88 (0.14) 3. 0.90 (0.13) 4. 0.85 (0.17) 5. 0.77 (0.13) 6. 1.0 (0.01) |
| **NN** | 1. 16 scenarios 2. 0.89 (0.16) 3. 0.89 (0.19) 4. 0.88 (0.15) 5. 0.78 (0.15) 6. 0.98 (0.05) | 1. 8 scenarios 2. 0.88 (0.14) 3. 0.87 (0.18) 4. 0.86 (0.20) 5. 0.78 (0.14) 6. 0.98 (0.03) | 1. 14 scenarios 2. 0.89 (0.14) 3. 0.88 (0.17) 4. 0.88 (0.15) 5. 0.80 (0.10) 6. 0.99 (0.02) |
| **SVR Linear** | 1. 15 scenarios 2. 0.86 (0.16) 3. 0.88 (0.19) 4. 0.82 (0.15) 5. 0.75 (0.15) 6. 0.98 (0.01) | 1. 11 scenarios 2. 0.85 (0.15) 3. 0.89 (0.17) 4. 0.79 (0.14) 5. 0.74 (0.13) 6. 0.96 (0.07) | 1. 13 scenarios 2. 0.87 (0.15) 3. 0.89 (0.18) 4. 0.83 (0.14) 5. 0.77 (0.13) 6. 0.98 (0.06) |
| **SVR RBF** | 1. 16 scenarios 2. 0.86 (0.17) 3. 0.89 (0.17) 4. 0.83 (0.18) 5. 0.73 (0.15) 6. 0.98 (0.01) | 9 scenarios   1. 0.85 (0.15) 2. 0.89 (0.16) 3. 0.79 (0.14) 4. 0.73 (0.14) 5. 0.97 (0.04) | 1. 13 scenarios 2. 0.87 (0.16) 3. 0.89 (0.17) 4. 0.83 (0.17) 5. 0.75 (0.14) 6. 0.99 (0.02) |

**Table S1** Summary of results for the ML methods. i) Number of scenarios in which the method achieved a perfect selection of the covariate. ii) The average F1 score with standard deviation in brackets for all scenarios, (iii) simple scenarios (1-2), (iv) complex scenarios (5-6), (v) scenarios with small effect size (a, c, e), and (vi) scenarios with high effect size (b, d, f).

| **PMX approaches** | |
| --- | --- |
| **SCM** | - - - 1. 0 scenario       2. 0.84 (0.18)       3. 0.83 (0.24)       4. 0.84 (0.15)       5. 0.71 (0.17)       6. 0.98 (0.01) |
| **SCM_TR** | - - - 1. 0 scenario       2. 0.75 (0.18)       3. 0.73 (0.27)       4. 0.74 (0.15)       5. 0.61 (0.17)       6. 0.89 (0.03) |
| **COSSAC** | - - - 1. 0 scenario       2. 0.79 (0.16)       3. 0.82 (0.22)       4. 0.76 (0.13)       5. 0.70 (0.15)       6. 0.93(0.04) |
| **COSSAC_TR** | - - - 1. 0 scenario       2. 0.72 (0.18)       3. 0.73 (0.26)       4. 0.72 (0.16)       5. 0.61 (0.16)       6. 0.87 (0.04) |
| **LASSO** | - - - 1. 0 scenario       2. 0.65 (0.18)       3. 0.70 (0.23)       4. 0.58 (0.16)       5. 0.63 (0.18)       6. 0.67 (0.16) |

**Table S2** Summary of results for the PMX approaches. i) Number of scenarios in which the method achieved a perfect selection of the covariate. ii) The average F1 score with standard deviation in brackets for all scenarios, (iii) the small scenarios (1-2), (iv) the large scenarios (5-6), (v) scenarios with small effect size (a, c, e), and (vi) scenarios with high effect size (b, d, f). TR: True.

|  | ***p-*value (forward, backward)** | | | | | | | | |
| --- | --- | --- | --- | --- | --- | --- | --- | --- | --- |
| **Scenario** | **(0.05, 0.01)** | **(0.05, 0.05)** | **(0.05, 0.04)** | **(0.05, 0.03)** | **(0.05, 0.02)** | **(0.05, 0.01)** | **(0.1, 0.01)** | **(0.15, 0.01)** | **(0.2, 0.01)** |
| **1a** | 0.31 | 0.309 | 0.309 | 0.309 | 0.309 | 0.175 | 0.443 | 0.519 | 0.533 |
| **1b** | 0.43 | 0.427 | 0.427 | 0.427 | 0.427 | 0.191 | 0.513 | 0.514 | 0.565 |
| **5a** | 0.6 | 0.585 | 0.593 | 0.594 | 0.6 | 0.597 | 0.594 | 0.594 | 0.594 |

**Table S3** F1 scores obtained with SCM in the three worst cases for pairs of p-values different from the one reported in the first columns, which was the default and used in all scenarios.

| **ML approaches** | | | |
| --- | --- | --- | --- |
| **Method\Selection** | **Top-M** | **Order of Importance** | **Minimum of Importance** |
| **RF** | - 7a: 0.38 - 7b: 0.43 | - 7a: 0.5 - 7b: 0.48 | - 7a: 0.64 - 7b: 0.6 |
| **NN** | - 7a: 0.5 - 7b: 0.51 | - 7a: 0.64 - 7b: 0.61 | - 7a: 0.63 - 7b: 0.60 |
| **SVR Linear** | - 7a: 0.34 - 7b: 0.46 | - 7a: 0.39 - 7b: 0.47 | - 7a: 0.46 - 7b: 0.47 |
| **SVR RBF** | - 7a: 0.29 - 7b: 0.37 | - 7a: 0.35 - 7a: 0.38 | - 7a: 0.36 - 7b: 0.38 |
| **PMX approaches** | | | |
| **SCM** | - 7a: 0.27 - 7b: 0.45 | | |
| **SCM_TR** | - 7a: 0.2 - 7b: 0.35 | | |
| **COSSAC** | - 7a: 0.34 - 7b: 0.36 | | |
| **COSSAC_TR** | - 7a: 0.29 - 7b: 0.14 | | |

**Table S4** Summary of the average F1 scores of the ML methods and PMX methods for scenarios 7a and 7b. For ML methods, results with the three considered approaches (top-M, order of importance, minimum of importance) are shown. TR: True.

| **ML approaches** | | | |
| --- | --- | --- | --- |
| **Method\Selection** | **Top-M** | **Order of Importance** | **Minimum of Importance** |
| **RF** | 0.66 | 0.65 | 0.72 |
| **NN** | 0.66 | 0.71 | 0.69 |
| **SVR Linear** | 0.65 | 0.69 | 0.65 |
| **SVR RBF** | 0.58 | 0.6 | 0.57 |
| **PMX approaches** | | | |
| **SCM** | 0.62 | | |
| **SCM_TR** | 0.55 | | |
| **Linear SCM** | 0.62 | | |
| **Linear SCM_TR** | 0.57 | | |
| **GAM** | 0.45 | | |
| **COSSAC** | 0.53 | | |
| **COSSAC** | 0.46 | | |

**Table S5** Summary of the average F1 scores of the ML methods and PMX methods including GAM and linearization-based SCM for scenarios 6e with additive residual error. For ML methods, results with the three considered approaches (top-M, order of importance, minimum of importance) are shown. TR: True.
